# Using gene expression databases for classical trait QTL candidate gene discovery in the BXD recombinant inbred genetic reference population: Mouse forebrain weight

**DOI:** 10.1186/1471-2164-9-444

**Published:** 2008-09-25

**Authors:** Lu Lu, Lai Wei, Jeremy L Peirce, Xusheng Wang, Jianhua Zhou, Ramin Homayouni, Robert W Williams, David C Airey

**Affiliations:** 1 Jiangsu Key Laboratory of Neuroregeneration, Nantong University, PR China; 2 Department of Anatomy and Neurobiology, University of Tennessee Health Sciences Center, Memphis, TN, 38103, USA; 3 Department of Pharmacology, Vanderbilt University School of Medicine, Nashville, TN, 37232, USA; 4 Department of Pathology and Laboratory Medicine, University of Tennessee Health Sciences Center, Memphis, TN, 38103, USA; 5 Department of Neurology, University of Tennessee Health Sciences Center, Memphis, TN, 38103, USA; 6 Department of Medicine, University of Massachusetts Medical School, Worcester, MA, 01605, USA

## Abstract

**Background:**

Successful strategies for QTL gene identification benefit from combined experimental and bioinformatic approaches. Unique design aspects of the BXD recombinant inbred line mapping panel allow use of archived gene microarray expression data to filter likely from unlikely candidates. This prompted us to propose a simple five-filter protocol for candidate nomination. To filter more likely from less likely candidates, we required candidate genes near to the QTL to have mRNA abundance that correlated with the phenotype among the BXD lines as well as differed between the parental lines C57BL/6J and DBA/2J. We also required verification of mRNA abundance by an independent method, and finally we required either differences in protein levels or confirmed DNA sequence differences.

**Results:**

QTL mapping of mouse forebrain weight in 34 BXD RI lines found significant association on chromosomes 1 and 11, with each C57BL/6J allele increasing weight by more than half a standard deviation. The intersection of gene lists that were within ± 10 Mb of the strongest associated location, that had forebrain mRNA abundance correlated with forebrain weight among the BXD, and that had forebrain mRNA abundance differing between C57BL/6J and DBA/2J, produced two candidates, *Tnni1 *(troponin 1) and *Asb3 *(ankyrin repeat and SOCS box-containing protein 3). Quantitative RT-PCR confirmed the direction of an increased expression in C57BL/6J genotype over the DBA/2J genotype for both genes, a difference that translated to a 2-fold difference in Asb3 protein. Although Tnni1 protein differences could not be confirmed, a 273 bp indel polymorphism was discovered 1 Kb upstream of the transcription start site.

**Conclusion:**

Delivery of well supported candidate genes following a single quantitative trait locus mapping experiment is difficult. However, by combining available gene expression data with QTL mapping, we illustrated a five-filter protocol that nominated *Asb3 *and *Tnni1 *as candidates affecting increased mouse forebrain weight. We recommend our approach when (1) investigators are working with phenotypic differences between C57BL/6J and DBA/2J, and (2) gene expression data are available on  that relate to the phenotype of interest. Under these circumstances, measurement of the phenotype in the BXD lines will likely also deliver excellent candidate genes.

## Background

Strategies for discovering the genetic polymorphism responsible for an identified quantitative trait locus (QTL) generally follow two paths. One path involves generating additional experimental mapping populations to narrow an initial, wide QTL support interval [1]. For example, Yalcin et al. [2], used outbred mice and a QTL-knockout interaction test to identify *Rgs2 *as the gene underlying an anxiety phenotype. The other path involves making use of bioinformatic tools and archival data to better nominate candidate genes within a QTL support interval [3,4]. For example, Flint and colleagues review and apply a hypothesis of human and mouse sequence conservation that may aid QTL gene or polymorphism discovery [5-7]. The combination of approaches should facilitate polymorphism identification, and more rapidly.

The BXD, an increasingly popular tool for mouse complex trait genetics, are a panel of recombinant inbred lines derived by inbreeding progeny from a C57BL/6J × DBA/2J F2 intercross [8]. Because the genetic variation in BXD mice is between line rather than between animal, the BXD panel is a genetic reference population (a retrievable resource). This useful design allows mapping of QTL affecting a trait by correlating variation among lines to a set of genetic markers available in online databases. In addition, there is a growing collection of databases of gene expression for the BXD lines at  that provides an additional level of genome wide interrogation [9-11]. We believe that this provides an opportunity to combine experimental and bioinformatic approaches from a single BXD mapping experiment to very rapidly and efficiently nominate candidate genes. We propose and validate a five-filter protocol for this purpose, as follows.

1. List all genes within 10 Mb of the point of maximum likelihood of the QTL map location.

2. List all genes that differ in mRNA abundance between the parental lines C57BL/6J and DBA/2J.

3. List all genes for which mRNA abundance correlates with the target trait among the BXD lines.

4. Identify the genes at the intersection of all three filters and verify mRNA abundance of these genes in parent lines and BXD lines by an independent method.

5. For the genes that remain after the first four filters, demonstrate differences in their DNA sequence, or in the levels of the proteins that they encode.

This protocol proved highly effective in identifying two strong candidates in a validation study targeting forebrain weight, and we believe it can serve with equal effectiveness for other traits mapped in the BXD lines, and eventually for traits mapped in the Collaborative Cross (CC) under development by the Complex Trait Consortium [12].

Brain size is a trait of historical and evolutionary interest [13], and is on occasion the focus of unpopular hypotheses related to normal function and individual differences [14]. Perhaps of greater concern, however, is that the metric is also of importance to biomedical science, where a number of human disorders present developmental alterations in total or component brain size measures (e.g., [15,16]). Forebrain weight in mice, as in humans, is a surrogate measure of numerous and aggregate developmental processes related to cell division, migration, death, and differentiation [17]. Given that QTL for human disorders and mouse models thereof have been mapped to homologous chromosomal regions [4], forebrain weight analysis in mice may advance our understanding of developmental mechanisms that contribute to brain size and that have clinical relevance to human health and well-being.

## Results

### Forebrain weight QTLs

Forebrain weight in 34 BXD RI lines and the two parental strains C57BL/6J and DBA/2J ranged from 260 to 352 mg, with a mean (SD) of 304 mg (20 mg). Forebrain weight adjusted for variation in body weight, age, sex, and brain weight other than forebrain (see Methods), was expectedly reduced in total variation (SD = 17.5 mg), but otherwise retained the approximate normal distribution of the unadjusted forebrain weight, and was well correlated with unadjusted weight (*r *= 0.959).

**Figure 1:**
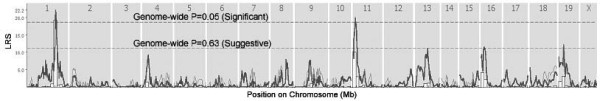
**Genome scan likelihood ratio statistic plot**. Depicted is a whole genome scan for QTLs affecting mouse forebrain weight. X axis depicts 19 autosomes and X chromosome. The Y axis is the likelihood ratio statistic from a single QTL model. Two QTLs, on chromosomes 1 and 11, are significant at a multiple test corrected permutation threshold as shown.

**Figure 2:**
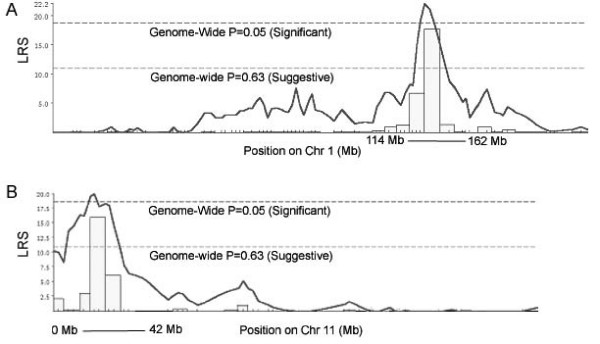
**Chromosome 1 and 11 likelihood ratio statistic plots**. Interval mapping plots of chromosomes 1 and 11, showing more detail of Figure 1. 2 LOD support intervals are shown in Mb on the X axis.

Simple interval mapping of adjusted forebrain weight revealed two QTLs on chromosome 1 (*Fbrwt1*) and 11 (*Fbrwt11*) with likelihood ratio statistics (LRSs) above a significance threshold determined by 10,000 permutations of the data (Figures 1, 2). Each C57BL/6J allele was estimated to increase adjusted forebrain weight by 10 mg, somewhat more than half a standard deviation in effect size. A pairwise scan using the DIRECT algorithm for epistasis detection [18] implemented at  did not discover significant interaction between these or other loci.

#### Filter 1: Candidates near to the forebrain weight QTLs

A total of 240 genes were found within ten megabases of the highest LRS for the QTLs discovered on chromosomes 1 and 11, with 138 genes between 125 Mb and 145 Mb on chromosome 1, and 102 genes between 20 and 40 Mb on chromosome 11 (see Additional file 1).

#### Filter 2: Candidates with microarray gene expression correlated with forebrain weight

A total of 329 genes had expression levels that correlated significantly with adjusted forebrain weight (*p *< 0.05) across 32 BXD RI lines. Of these, 9 identified genes were within 10 Mb of the QTL *Fbrwt1 *and 6 were within 10 Mb of the QTL *Fbrwt11 *(see Additional file 2).

#### Filter 3: Candidates with microarray gene expression differences in C57BL/6J and DBA/2J

**Figure 3:**
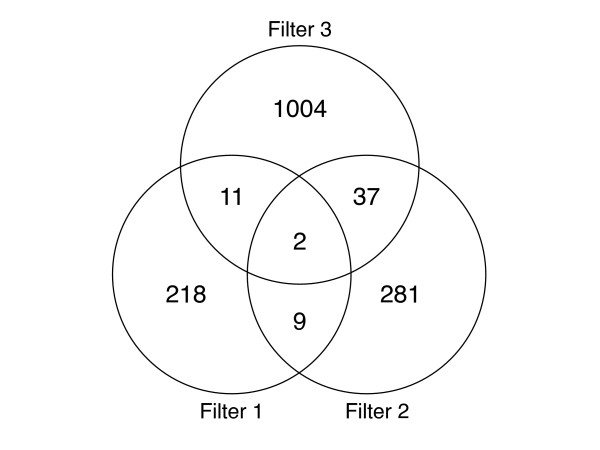
**Venn diagram for the intersection of filters 1, 2, and 3**. Filter 1 is the set of 240 genes residing within 10 Mb of the peak LRS for QTLs on Chr 1 and 11 as in Figure 2. Filter 2 is the set of 329 genes with expression levels correlated with forebrain weight. Filter 3 is the set of 1054 genes with expression differences between C57BL/6J and DBA/2J forebrain tissue. As shown, two genes are shared by all three filters. See "Additional files" for the actual gene lists.

A total of 1,054 genes were significantly different comparing three U74Av2 microarrays with pooled C57BL/6J forebrain RNA against three U74Av2 microarrays with pooled DBA/2J forebrain RNA by t-test (*p *< 0.05) (see Additional file 3). Of the 11 genes retained by filters 1 and 2, two genes were also shown to have differential gene expression between the C57BL/6J and DBA/2J (Figure 3). These were *Tnni1*, or troponin 1, on chromosome 1, and *Asb3*, or ankyrin repeat and SOCS box-containing protein 3, on chromosome 11. For both genes, the C57BL/6J allele increased expression, with a 3 fold change for *Tnni1 *and a 2 fold change for *Asb3*.

Because multiple data sets of forebrain microarray gene expression are available at , we also looked at the expression of *Tnni1 *and *Asb3 *in one additional microarray data set. We compared 4 C57BL/6J and 4 DBA/2J Affymetrix M430 chips, by t-test. This data set confirmed that C57BL/6J again had higher transcript abundance for *Tnni1 *(*p *= 0.004) and *Asb3 *(*p *= 0.007).

**Figure 4:**
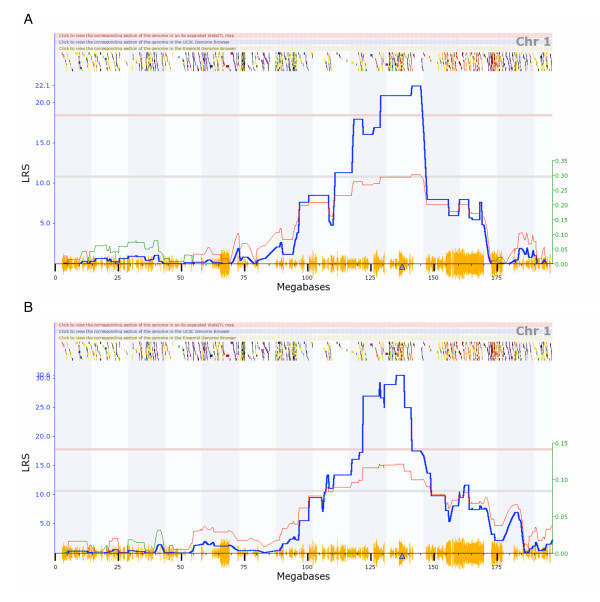
**eQTL interval mapping of Tnni1**. eQTL mapping of *Tnni1 *transcript abundance across BXD lines in two independent databases, based on (A) Affymetrix U74Av2 and (B) M430 microarray platforms. In both databases, significant association overlies the location of *Tnni1*, suggesting a potential *cis*-eQTL. This figure is directly from . The blue curve is the LRS trace; the red curve follows the right Y axis (effect size in standard deviations). The red horizontal line indicates genome wide significance. The triangle on the baseline is the position of *Tnni1*. The orange chatter along the X axis indicates the density of SNPs present in the BXD. The multicolored chatter along the top of the graph are hyperlinks to sites with additional genetic and sequence information (when a graph is viewed live at ).

**Figure 5:**
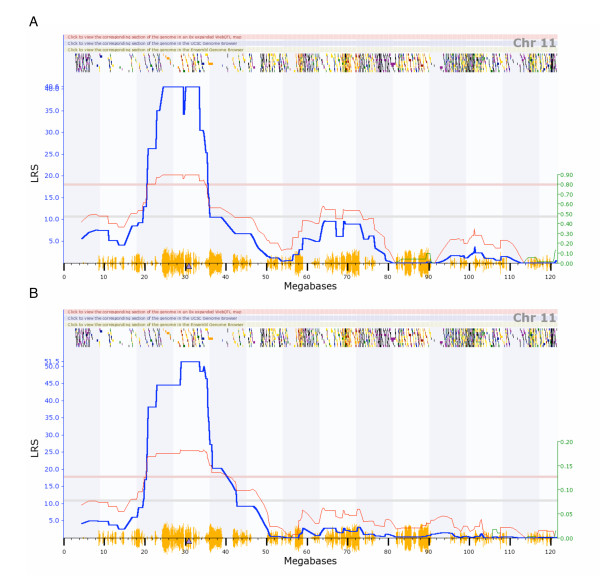
**eQTL interval mapping of Asb3**. eQTL mapping of *Asb3 *transcript abundance across BXD lines in two independent databases, based on (A) Affymetrix U74Av2 and (B) M430 microarray platforms. In both databases, significant association overlies the location of *Asb3*, suggesting a potential *cis*-eQTL. This figure is directly from . The blue curve is the LRS trace; the red curve follows the right Y axis (effect size in standard deviations). The red horizontal line indicates genome wide significance. The triangle on the baseline is the position of *Asb3*. The orange chatter along the X axis indicates the density of SNPs present in the BXD. The multicolored chatter along the top of the graph are hyperlinks to sites with additional genetic and sequence information (when a graph is viewed live at ).

When either *Tnni1 *or *Asb3 *transcript abundance is mapped to locate QTLs controlling expression, both show strong evidence of control by *cis*-eQTLs (Figure 4, 5). *Tnni1 *transcript abundance maps to the chromosomal location of itself with an LRS of 25.2 (U74Av2 data set) and 30.6 (M430 data set). *Asb3 *transcript abundance maps to the chromosomal location of itself with an LRS of 36.1 (U74Av2 data set) and 51.5 (M430 data set).

In summary, candidate gene *Tnni1 *lies near (135.6 Mb) to the position of highest LRS for the *Fbrwt1 *QTL (142 Mb), has gene expression variation in the BXD mapping population that correlates with the mapped phenotype (Spearman's rho = 0.44, *p *= 0.01), and also has gene expression that differs between BXD parental inbred strains C57BL/6J and DBA/2J (t = 5.99, *p *= 0.0039; U74Av2 chips). Similarly, candidate gene *Asb3 *lies near (31 Mb) to the position of the highest LRS for the *Fbrwt11 *QTL (30 Mb), has gene expression that correlates with forebrain weight (Spearman's rho = 0.50, *p *= 0.003), and also has gene expression difference between C57BL/6J and DBA/2J (t = 5.32, *p *= 0.007; U74Av2 chips).

#### Filters 4 and 5: Confirmation by RT-PCR (Tnni1 and Asb3) and Western blot (Asb3)

**Figure 6:**
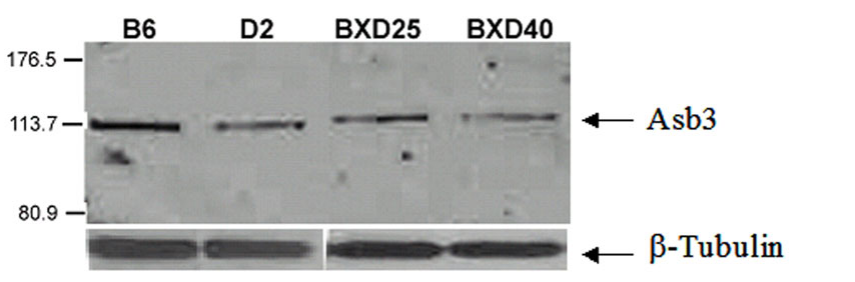
**Western blot of Asb3 in C57BL/6J, DBA/2J, BXD25, and BXD40**. C57BL/6J (B6) and BXD25 show elevated Asb3 protein abundance relative to DBA/2J (D2) and BXD40. The uniform intensity of the *β*-tubulin staining across all samples validates the consistency of the total protein loaded for each strain.

To verify gene expression differences with an independent method, reverse transcriptase PCR was performed on C57BL/6J and DBA/2J forebrain total RNA. Both *Tnni1 *and *Asb3 *showed greater transcript abundance in C57BL/6J relative to DBA/2J, with *Tnni1 *3.3-fold different, and *Asb3 *2.5-fold different. Two BXD strains with high (BXD25) and low (BXD40) microarray expression for *Asb3 *were also analyzed by RT-PCR, indicating a 8.4 fold increase in mRNA expression in BXD25 forebrain compared to BXD40 forebrain. Finally, using an antibody against *Asb3 *protein, Western blot analysis showed a 2.3 fold increase in BXD25 forebrain compared to BXD40 forebrain (Figure 6).

### Tnni1 promoter sequence analysis

**Figure 7:**
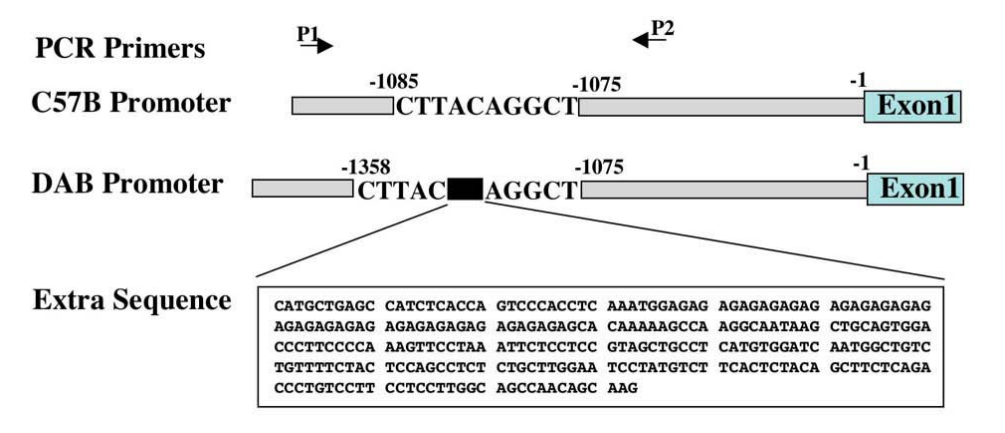
**Deletion polymorphism in DBA/2J Tnni1**. An extra 273 bp was present in *Tnni1 *promoter of DBA/2J mice but not in C57BL/6J mice. P1 and P2 are primers that were used to amplify the 401 bp fragment of mice *Tnni1 *promoter. The extra 273 bp is located 1080 bp upstream of transcription start site (beginning of Exon 1).

Although we were unable to verify differences in *Tnni1 *protein abundance (see Methods), we were able to discover a 273 bp insertion located 1080 bp upstream of the *Tnni1 *transcription start site, that was present in DBA/2J mice, but absent in C57BL/6J mice (Figure 7). C57BL/6J sequences confirmed those published in Genbank for the *Tnni1 *promoter. Bioinformatic analysis of the indel polymorphism suggests the presence of multiple transcription factor binding sites (see Additional file 4).

## Discussion

### Synopsis

Using a five-filter protocol for QTL candidate gene nomination that combines classical trait QTL mapping with gene expression data in BXD mice, we provide convergent evidence that nominates troponin 1 (*Tnni1*, Chr 1) and ankyrin repeat and SOCS box-containing protein 3 (*Asb3*, Chr 11) as candidate genes for QTLs affecting mouse forebrain weight variation, with C57BL/6J alleles conferring increased weight over DBA/2J alleles. To nominate these candidate genes, we required candidate genes near to the forebrain weight QTL to have correlated mRNA abundance among the BXD lines as well as a difference between the parental lines C57BL/6J and DBA/2J. We further required verification of mRNA abundance by an independent method, and finally we required either differences in protein levels or confirmed DNA sequence differences. *Tnni1 *and *Asb3 *met each of these requirements.

### Tnni1 and Asb3 in brain development

*Asb3 *encodes a member of the ankyrin repeat and SOCS box containing proteins [19]. Ankyrin repeats are a common motif involved in molecular recognition via protein-protein interactions. The SOCS box found in the Asb family of proteins targets suppressor of cytokine signaling (SOCS) proteins with the elongin B/C complex and can be involved in targeting for degredation. *Asb3 *is widely expressed, including high levels of expression in the brain and mediates ubiquitination and degredation of tumor necrosis factor receptor II (TNF-R2). *Asb3 *protein is thus also involved in inhibition of TNF-R2-mediated Jun N-terminal protein kinase (JNK) activation. JNK is involved in cortical neuron migration [20] and may be involved in signaling cell survival in developing forebrain [21] which suggests a role for *Asb3 *in forebrain development.

*Tnni1 *encodes the slow-twitch muscle isoform of Troponin I [22], the inhibitory subunit of the troponin complex. Although we could not verify the effect of *Tnni1 *transcript differences on protein levels, we did discover a 273 base pair indel polymorphism upstream of the transcription start site of *Tnni1 *in which we found bioinformatic evidence for transcription factor binding sites. Without testing a broader sample of inbred lines for the presence of the indel and *Tnni1 *expression, it remains unknown if this polymorphism is an interfering insertion, reducing DBA/2J *Tnni1 *transcription. Nonetheless, a working hypothesis is that the discovered indel is a *Tnni1 *promoter variant underlying the *cis*-eQTL for *Tnni1 *mRNA abundance and the QTL for forebrain weight. *Tnni1 *is typically expressed in skeletal muscle [23,24], and functions to prevent actin-myosin interaction, but it is also considered a cytoskeletal element in some neurons [25]. We demonstrated that *Tnni1 *mRNA is also found in the mouse brain, and we suggest it may have alternative functions in CNS tissue, perhaps during development. This is particularly interesting because of recent work on mutations in other cytoskeletal element genes and their roles in human brain size and microcephaly [26].

### Previous study of brain size in the BXD

An earlier paper by Belknap et al., [27] reported two QTL affecting brain to body weight ratios on chromosomes 11 (9 cM) and 17 (25–40 cM) in a sample of 20 BXD RI lines. The QTL reported on Chr 11 is in the same proximal location as *Fbrw11 *reported above. As defined, our forebrain weights included midbrain and forebrain (telencephalon, diencephalon, and mesencephalon), but excluded olfactory bulbs and the brain stem (metencephalon and myelencephalon). We also used linear regression to control body weights rather than ratios [27,28], and unlike Belknap et al., [27], we included both sexes, and used a wider range of ages. Nonetheless, the genetic correlation between our measures and those reported more than 15 years ago in Table 1 in [27] for total brain weight to body weight ratios for male mice is *r *= 0.58 (*p *= 0.0068) [29]. It is therefore possible that the same genetic signal (candidate *Asb3*) underlies both QTLs. A more recent study on neocortex volume and remaining brain volume among BXD lines suggests that the genetic signal is mostly expressed in neocortex variation. Beatty and Laughlin [30] reported a QTL for neocortex volume in the same location as *Fbrw11*; noncortical brain volume did not reach statistical significance on proximal Chr 11 after genome-wide multiple test correction [30]. The correlation between our forebrain weights and the neocortex volumes was *r *= 0.73 (*p *< 0.0001).

### Advantages and disadvantages of the five-filter protocol

The literature is replete with reviews describing a status quo of relative ease of QTL identification and great difficulty discovering the underlying polymorphism (e.g., see [1]). Indeed, without bioinformatic support, the purely experimental path to polymorphism identification is effortful, long, and without guaranteed success. A key pillar of the protocol we describe is use of the BXD genetic reference population and the availability of gene expression data on each BXD line. The list and specificity of traits for which BXD gene expression is available is growing rapidly, and already contains different organs as well as specific cell types . Multiple conditions are also coming online (e.g., developmental time (cerebellum) or environmental manipulations (stress, alcohol)). It is not strictly necessary for the gene expression database to exactly match the measured trait for our five-filter protocol to be applied. Behavioral phenotypes could reasonably be correlated to brain expression databases (e.g., *trait *anxiety but perhaps not *state *anxiety). Certainly, the correlations would become more relevant with a more specific expression database (e.g., amygdala), and cases can be conjectured for which our approach would be uninterpretable (e.g., G × E QTLs using only baseline gene expression databases). In such cases, our suggested protocol is not flawed, but the available resources would need generating (an expensive proposition). In the present study, for example, we used adult gene expression to make inferences about development of forebrain weight. This will work in cases where developmental switches are left on (e.g., [31,32]) but will fail with temporally limited gene expression differences (e.g., [33,34]). More complete results will come from application of the five filters to a developmental forebrain gene expression database.

Some of the strengths of our protocol are also disadvantages. The use of BXD mice necessarily limits the number of genomes investigated to only two common inbred strains (C57BL/6J and DBA/2J). Unfortunately, there is a dearth of large mouse recombinant inbred line panels, although this may change in the future [12]. Our results, for example, could be extended by validating brain weight as measured in the current BXD RI lines [35] to the more recently developed, advanced intercross derived BXD RI lines [8]. Finally, nominating genes that act through gene expression also presents an obvious weakness, by missing gene variants that cause null or poorly trafficked proteins, for example. Despite these limitations, the methods we illustrate warrant careful consideration by those working with phenotypic differences between C57BL/6J and DBA/2J.

## Conclusion

Delivery of well supported candidate genes following a single quantitative trait locus mapping experiment is difficult. However, by combining available gene expression data with QTL mapping, we illustrated a five-filter protocol that nominated *Asb3 *and *Tnni1 *as candidates affecting increased mouse forebrain weight. We recommend our approach when (1) investigators are working with phenotypic differences between C57BL/6J and DBA/2J, and (2) gene expression data are available on  that relate to the phenotype of interest. Under these circumstances, measurement of the phenotype in the BXD lines will likely also deliver excellent candidate genes.

## Methods

### Ethics

All experimental procedures were performed in accordance with (1) the *Guidelines for the Care and Use of Laboratory Animals *published by the National Institutes of Health (publication 86-23) and (2) the University of Tennessee Health Sciences Center Animal Care and Use Committee (protocol number 680).

### BXD RI mice

Separate cohorts of 34 BXD recombinant inbred (RI) lines as well as inbred strains C57BL/6J (B6) and DBA/2J (D2) were used for QTL mapping and gene expression. BXD RI lines were generated by Taylor and colleagues [35] from C57BL/6J and DBA/2J parental strains in the mid-1970s (BXD1 through 32) and 1990s (BXD33 through 42); additional so-called "Williams" lines were generated recently by our group [8]. RI strains are fully inbred lines derived from brother-sister matings starting from an F2 intercross. Although forebrain weight data are now available for both the original "Taylor" BXD mice and the "Williams" BXD mice, we focus only on the "Taylor" BXD mice. Not all of the "Williams" BXD mice are fully inbred, and some lines retain a small amount of heterozygosity that may affect forebrain weight though dominance mechanisms we do not account for in this paper.

### Husbandry and age

Mice were housed at 20 to 24°C on a 14/10 h light/dark cycle in a specific pathogen-free (SPF) facility at the University of Tennessee. All animals were fed 5% fat Agway Prolab 3000 (Agway Inc., Syracuse, NY) rat and mouse chow. The average age of BXD/Ty animals at the time of sacrifice was 82 days with a range of 21–763; age did significantly predict forebrain weight and was considered along with other factors in our forebrain weight model below.

### Tissue fixation

Mice were deeply anesthetized with Avertin (1.25% 2,2,2-tribromoethanol and 0.8% tert-pentyl alcohol in water, 0.5–1.0 intraperitoneal injection). Next, they were transcardially perfused with 0.1 M phosphate buffered saline followed by 4% paraformaldehyde in 0.1 M phosphate buffer. Tissues were stored in fixative thereafter.

## Dissection

Forebrain weight was defined to include all brain rostral of the metencephalon, excluding olfactory bulbs. The forebrain was dissected free of the olfactory bulbs by cutting across the ventral midline at the waist of the olfactory peduncle behind the ventral-caudal end of the glomerular surface of the bulb, and was dissected free of the hindbrain by cutting at the junction of midbrain and pons. The brain was rolled quickly in tissue paper and immediately weighed to the nearest 0.1 mg. The forebrain dissection thus includes most of the forebrain and midbrain, bilaterally, but excludes the olfactory bulbs, retinas, and the posterior pituitary (all formally part of forebrain).

### Microarrays

The gene expression data set used for these analyses was selected from a larger set that has been previously described [11]. There is a link to extensive metadata describing the samples and sample processing on GeneNetwork . Briefly, tissues were dissected from BXD animals (both sexes, aged 8, 20, or 52 weeks) in 32 of the same strains (but different animals) as for the forebrain weight analysis, but using unfixed tissue. Total RNA was extracted and labeled according to Affymetrix protocols and hybridized with Affymetrix U74Av2 microarrays . A total of 2–4 littermates were dissected and equal amounts of tissue were combined (pooled) for hybridization to each array. A total of 100 arrays were used. Array data were normalized using the MAS5 algorithm from Affymetrix. For a subset of confirmation analyses, we accessed a newer Affymetrix M430 microarray dataset (described at ).

### Modeling forebrain weight

The number of BXD mice used to collect forebrain weights was 386, with an average of 11 mice measured per strain (minimum, 25%, median, 75%, maximum: 3, 7, 10, 15, 21). Because our forebrain weight measurements were not taken from a population balanced for important covariates, we used multiple regression to fit effects of age, body weight, sex, and non-forebrain brain weight (weight of total brain after the weight of the forebrain was substracted). Residual forebrain weights are available on  (Trait 10701, standardized to the mean forebrain weight by addition of the average forebrain weight by strain) as are simple raw trait averages by strain (Trait 10699).

### Genotyping and QTL mapping

QTL and eQTL mapping was performed using GeneNetwork  and a standardized set of 3795 genotyped markers (mapping algorithm and genotypes described at ; genotypes downloadable as a text file from ). Residuals from the model described above (Trait 10701) were simple interval mapped using a modified Haley-Knott algorithm [36,37], weighted by the within strain variances. Genome-wide significance was calculated by comparing the best likelihood ratio statistic of the original data set with the distribution of highest LRS computed for 10,000 permutations.

eQTL mapping is QTL mapping of gene transcript abundance, generally measured by microarray. eQTLs can be classified as either *cis*-eQTLs, that map to the same location of gene encoding the transcript being mapped, or *trans*-eQTLs, that map to locations other than the gene encoding the mapped transcript. *Cis*-eQTLs are suggestive of a polymorphism in the gene promoter.

### Five filters for candidate gene discovery

Selection criteria for candidate genes included five filters. The *first *filter required a candidate gene to be located near to the mapped forebrain QTL, within 10 million bases from the genetic marker with the highest LRS resulting from simple interval mapping. Physical locations of genes in the BXD are known, because the genomes of the parental inbred strains C57BL/6J and DBA/2J have been sequenced. Physical positions from the mm6 assembly of the mouse genome  were used with Genenetwork to generate lists of genes residing in or near to the QTLs. The *second *filter required a significant genetic correlation between forebrain microarray gene expression and forebrain weight among BXD strains. GeneNetwork can be used to rapidly estimate genetic correlation between BXD phenotypes [9,10]. We used GeneNetwork to correlate forebrain weight and gene expression from a dataset of 100 microarrays on 32 BXD RI lines (Spearman rho, alpha = 0.05). The *third *filter required a significant difference in forebrain microarray gene expression between the BXD parental inbred strains C57BL/6J and DBA/2J. Unpaired, equal variances t-tests were used to compare 3 and 3 Affymetrix U74Av2 microarrays (alpha = 0.05). Because gene microarray technology platforms change, we also verified candidate gene differences between C57BL/6J and DBA/2J using a newer microarray data set, based on the Affymetrix M430 A and B chips . The *fourth *filter required verification of gene expression differences by reverse transcriptase PCR (RT-PCR) in C57BL/6J and DBA/2J, and in a two BXD RI lines that had low and high transcript abundance by microarray. The *fifth *filter required protein differences by Western blot on genes verified by RT-PCR. Together, these five filters strongly nominate genes for classical trait QTLs that act by differences in gene expression.

The third filter, requiring a gene expression difference in the parental lines of the BXD, may be conservative, because an absence of a difference in the parental lines doesn't necessarily preclude heritability in the BXD. Shockley and Churchill [38] found more gene expression differences between A/J:C57BL/6J consomic lines than between the A/J and C57BL/6J parental inbred strains. One interpretation is that A/J and C57BL/6J carry compensating (epistatic) increaser and decreaser alleles that are segregated in the consomic lines. This has also been described in the BXD, when the RI lines have a range in phenotypic scores than is greater than ("transgresses") the difference between the C57BL/6J and DBA/2J parental inbred strains (e.g., [39]).

### Real-time PCR

Microarrays have been shown capable of yielding quantitative estimates of RNA levels [40,41]. However, it is generally accepted that differences benefit from verification with independent samples and methods. Also, in the present application of the short probe Affymetrix U74Av2 platform, it is possible that expression differences on the chip (but not *in vivo*) could arise from polymorphism between C57BL/6J and DBA/2J in Affymetrix probe sequences, because these were designed from C57BL/6J sequence information. BXD RI lines inheriting C57BL/6J alleles at such a location could in theory exhibit stronger hybridization than lines inheriting DBA/2J alleles. We used real-time PCR to verify expression differences, using duplicate samples for the parental strains C57BL/6J and DBA/2J, as well as duplicate samples for a high and low expressing BXD RIL, BXD40 and BXD25. Total RNA was isolated from whole brains using TRIzol reagent (Invitrogen, Carlsbad, CA). RT-PCR was performed on a SmartCycler (Cepheid, Sunnyvale, CA) using the AccessQuick RT-PCR system (Promega, Madison, WI), and SYBR green I (Molecular Probes, Eugene, Oregon) according to the manufacturer's instructions. The primers used to target mouse genes were (F is the forward primer, and R is the reverse): *Tnni1*, F: CAC CAG AGA GAT CAA GGA CC, R: TGT GCT TAG AGC CCA GTA GG; *Asb3*, F: TTT CAT CCA TCA GTT GCC AC, R: GCC TTG CTG GTT TCT CCA TC. Reverse transcription was performed at 48°C for 45 min and RT-PCR cycling parameters were as follows: denaturation at 95°C for 2 min followed by 35 cycles of amplification (94°C, 30 sec; 62°C, 30 sec). Product size was initially monitored by agarose gel electrophoresis and melting curves were analyzed to control for specificity of PCR reactions. The data on the target genes was normalized to the expression of the housekeeping gene *β*-actin and the relative units were calculated from a standard curve, plotting 3 different concentrations against the PCR cycle number at which the measured intensity reaches a fixed value (with a 10 fold increment equivalent to ~3.1 cycles).

### Western blot verification of protein abundance difference in Asb3

BXD25 and BXD40 mouse brains were lysed directly in radioimmunoprecipitation (RIPA) buffer for analysis of whole cell lysates. 50 *μ*g protein, calculated using a BCA (Bicinchoninic Acid) Protein Assay Kit (Pierce, Rockford, IL), were subjected to SDS-PAGE. Proteins were transferred to nitrocellulose membranes, immunoblotted with *Asb3 *specific antibodies (Santa Cruz Biotechnology, Inc., Santa Cruz, CA; antibody sc-19932) and visualized by enhanced chemiluminescence using the SuperSignal western blotting detection system (Pierce, Rockford, IL). The average intensity of bands was calculated using ImageJ . Unfortunately, the only available antibody for *Tnni1 *protein (Santa Cruz Biotechnology, Inc.) could not be made to work successfully in our lab, and we therefore only report results for *Asb3 *protein.

### Promoter sequence analysis

PCR fragments of *Tnni1 *were amplified from mice genomic DNA and subcloned into the pCR2.1 TA vector (Invitrogen). The sequences for primers P1 and P2 were: P1, 5' GAA TGG TAC CCC AGG TCG ACT TG 3' and P2, 5' AAG TCT GCT CTT CAC AGG TCA CA 3'. Sequencing was done by Macrogen (Rockville, MD).

The transcriptional start site (TSS) of *Tnni1 *was determined using the Database of Transcriptional Start Sites (DBTSS; ) [42]. Potential transcription factor-binding sites (TFBSs) were then identified using the TRANSFAC database and P-Match software by screening the upstream region of the *Tnni1 *indel sequence. All sites were found by the P-Match using the default parameters [43].

## Authors' contributions

LL designed experiments, and managed data collection. DCA and JLP prepared the manuscript. DCA and LL conducted statistical analysis. LW collected data and prepared figures. RH and JZ collected data. XW conducted bioinformatic analysis of promoter sequences. RW provided general guidance and grant support.

## Supplementary Material

Additional file 1 **List of genes within 10 Mb of Fbrwt1 or Fbrwt11.** Click here for file

Additional file 2 **List of forebrain weight correlations with gene expression.** Click here for file

Additional file 3 **List of gene expression differences between C57BL/6J and DBA/2J forebrain.** Click here for file

Additional file 4 **List of putative transcription factor binding sites in C57BL/6J and DBA/2J indel polymorphism.** Click here for file
